# Globally Distributed Arbuscular Mycorrhizal Fungi Associated With Invasive *Cinchona pubescens* on Santa Cruz Island, Galápagos

**DOI:** 10.1002/ece3.70462

**Published:** 2024-10-17

**Authors:** Paulo Herrera, Ingeborg Haug, Juan Pablo Suárez, Heinke Jäger

**Affiliations:** ^1^ Departamento de Ciencias Biológicas y Agropecuarias Universidad Técnica Particular de Loja, UTPL Loja Ecuador; ^2^ Evolutionary Ecology of Plants Eberhard‐Karls‐University Tübingen Tübingen Germany; ^3^ Charles Darwin Research Station Charles Darwin Foundation Santa Cruz Galápagos Ecuador; ^4^ Department of Ecology, Evolution, and Organismal Biology Brown University Providence Rhode Island USA; ^5^ Department of Ecology Technische Universität Berlin Berlin Germany

**Keywords:** ecosystem restoration, endangered species, Glomeromycota, invasive species, island colonization, OTUs

## Abstract

The presence of arbuscular mycorrhizal fungi (AMF) is essential for the survival and establishment of most plant species in nature. The reproductive success of invasive plant species in a particular habitat could also depend on these AM fungi. *Cinchona pubescens*, commonly known as quinine, is highly invasive on Santa Cruz Island, Galápagos, but at the same time severely endangered in its native range on mainland Ecuador due to overexploitation in the past. In this study, we aim at determining the AMF communities associated with *C. pubescens* at both locations to investigate whether the successful invasion of *C. pubescens* on Santa Cruz is related to its association with a particular community of AMF. For this, roots of *C. pubescens* trees were sampled at three sites, one site on Santa Cruz and two sites in the province of Loja, on mainland Ecuador. Communities of AM fungi were determined through the molecular cloning and sequencing of the 18S nrDNA gene and through the delimitation of Operational Taxonomic Units (OTUs), associated with the plant roots. We found 36 AMF OTUs associated with *C. pubescens*, most of them belonging to the genus *Glomus*. The highest richness of AMF OTUs was detected in samples from sites located on mainland Ecuador. The AMF communities between Santa Cruz and mainland Ecuador were significantly different, and only five OTUs were shared between both locations. Two dominant OTUs in *C. pubescens* from Santa Cruz were detected but no dominant OTUs from mainland Ecuador. Almost two thirds of the OTUs associated with *C. pubescens* had a wide global distribution. Our results suggest that the successful invasion of *C. pubescens* on Santa Cruz could have been facilitated by local generalist AMF and not by particular AMF. The observed generalist AMF from both locations could be important for conservation plans of restoring the endangered *C. pubescens* in the native forests on mainland Ecuador.

## Graphical abstract

Arbuscular mycorrhizal fungi (AMF) are crucial for plant survival and may influence the success of invasive species. This study investigated the AMF communities associated with the invasive *Cinchona pubescens* on Santa Cruz Island, Galápagos, and in its endangered native range in Ecuador. The AMF communities between Santa Cruz and mainland Ecuador were significantly different, suggesting that the successful invasion of *C. pubescens* on Santa Cruz could have been facilitated by local generalist AMF and not by particular AMF.

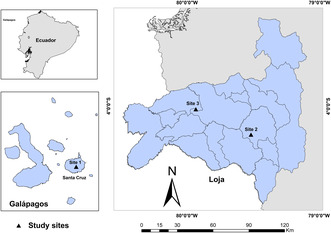

## 1 Introduction

Arbuscular mycorrhizal fungi (AMF) are distributed worldwide (Mosse, Stribley, and Letacon 1981) and are found in symbiotic association with more than 80% of the terrestrial plant species (Smith and Read 2008). All known AMF belong to the phylum Glomeromycota (Schüßler, Schwarzott, and Walker 2001; Tedersoo et al. 2018). The association with mycorrhizal fungi allows better absorption of soil nutrients and water by plants, improves the plant's tolerance to biotic and abiotic stresses, and stabilizes soil aggregates (Smith and Read 2008). In return, the plant provides photosynthetic products to the mycorrhizal fungi (Kiers et al. 2011).

Microbial communities and their interactions are known to be crucial biotic factors for maintaining key processes that regulate ecosystem functions (van der Heijden et al. 1998; van der Heijden, Bardgett, and van Straalen 2008). Early plant colonization processes, as is the case of isolated islands, are dominated by plant species without AMF dependence (Delavaux et al. 2019; Duchicela, Bever, and Schultz 2020). One example are the Galápagos Islands, which are well‐known for their unique flora and fauna, with a high degree of species endemism. In these young volcanic islands, located roughly 1000 km west of mainland South America, AMF colonize several plant species but with lower diversity compared to mainland ecosystems (Duchicela, Bever, and Schultz 2020). Distinct diversity, richness, or AMF communities, could influence the establishment of specific plant species at these sites (Lin, McCormack, and Guo 2015; van der Heijden et al. 1998). At the same time, the presence and composition of AMF communities in a particular ecosystem depend on multiple biotic and abiotic factors, highlighting the importance of the local habitat conditions and host plant species (van Geel et al. 2017). While debated, evidence suggests that ecosystem type plays a key role in shaping AMF communities (Öpik et al. 2013; Songachan and Kayang 2013; Wang et al. 2015), due to its abiotic filtering caused by some soil characteristics of the habitat, such as soil pH and phosphorus content (Moora et al. 2014; van Geel et al. 2017). If plant species introduced by humans to areas outside their natural distribution range can overcome biotic and abiotic factors acting as barriers for their establishment, these plant species have the potential to become invasive (Richardson and Pyšek 2012).

In Galápagos, the number of alien plant species has increased during recent years to at least 810 (Toral‐Granda et al. 2017). *Cinchona pubescens* Vahl, commonly known as “quinine” in English or “quinina” in Spanish, henceforth referred to as *C. pubescens*, is a tree belonging to the family Rubiaceae (Garmendia 2005). It is native to Costa Rica, Panama, Colombia, Venezuela, Peru, Bolivia, and Ecuador (Andersson 1998), and its bark was heavily harvested in the past due to its febrifuge and antimalarial properties (Crawford 2016). This was particularly the case in Ecuador, where populations of different *Cinchona* species were seriously decimated due to their overexploitation (Crawford 2016; Garmendia 2005). The province of Loja in Ecuador was the first to acquire fame as the most important source of *Cinchona* bark (Madsen 2002), and as a consequence, *C. pubescens* is now rare and endangered there (González‐Orozco, Guillén, and Cuvi 2023; Günter, Stimm, and Weber 2004). In contrast, in the Galápagos Islands, *C. pubescens* was introduced in the 1940s to the island of Santa Cruz, where it started spreading in the 1970s until it became invasive (Jäger 2015). Previous studies showed that the *C. pubescens* invasion caused a reduction in the diversity and abundance of native plant species, and changes in light, water, and soil nutrients regimes (Jäger, Kowarik, and Tye 2009; Jäger et al. 2013).

The AMF composition in the soil has a strong influence on plant community structure and diversity (O'Connor, Smith, and Smith 2002; van der Heijden et al. 1998), including in invasive plant species (Goodwin 1992; Pringle et al. 2009). Some studies indicated that AMF can facilitate the establishment of an invasive plant by increasing its competitive advantage over native species, as explained by the enhanced mutualist hypothesis (Reinhart and Callaway 2006; Sun and He 2010). On the other hand, successful invaders can become less reliant or even entirely independent on AMF in the new (nonnative) habitat, a shift to less dependence (Seifert, Bever, and Maron 2009).

Whether the AMF community associated with *C. pubescens* roots could have facilitated the successful invasion of *C. pubescens* on Santa Cruz in Galápagos is unknown. Studies have shown that *C. pubescens* is associated with AMF on Santa Cruz (Duchicela, Bever, and Schultz 2020; Schmidt and Scow 1986; Serrano 2013), but information on the diversity of the associated AMF is currently lacking. Likely, these AMF were already on Santa Cruz Island before the arrival of *C. pubescens*. Although the introduction pathway by which *C. pubescens* arrived on Santa Cruz is not documented, there is anecdotal information that it was brought in as saplings from mainland Ecuador, likely from the Loja region and likely with soil and therefore AMF attached to the roots (personal communication H. Jäger). Similarly, it is unclear how the association with AMF on Santa Cruz compares to the one of *C. pubescens* in its natural habitat on mainland Ecuador.

To understand how AMF may have influenced the successful invasion of *C. pubescens* on Santa Cruz Island, it is necessary to study the diversity of AMF associated with *C. pubescens* on Santa Cruz and mainland Ecuador. Therefore, we used molecular methods to compare the AMF communities associated with *C. pubescens* on Santa Cruz Island, Galápagos, and two sites in the Loja province, mainland Ecuador. For this, we addressed the following questions: (1) What is the richness of AMF associated with *C. pubescens*? (2) Does the richness of AMF differ between *C. pubescens* from Santa Cruz Island (introduced range) and the Loja province (native range)? and (3) Do AMF communities differ between *C. pubescens* inhabiting the two locations?

## 2 Materials And Methods

### 2.1 Study Sites

Roots of *C. pubescens* trees were sampled at one site in Galápagos and at two sites on mainland Ecuador.

**FIGURE 1:**
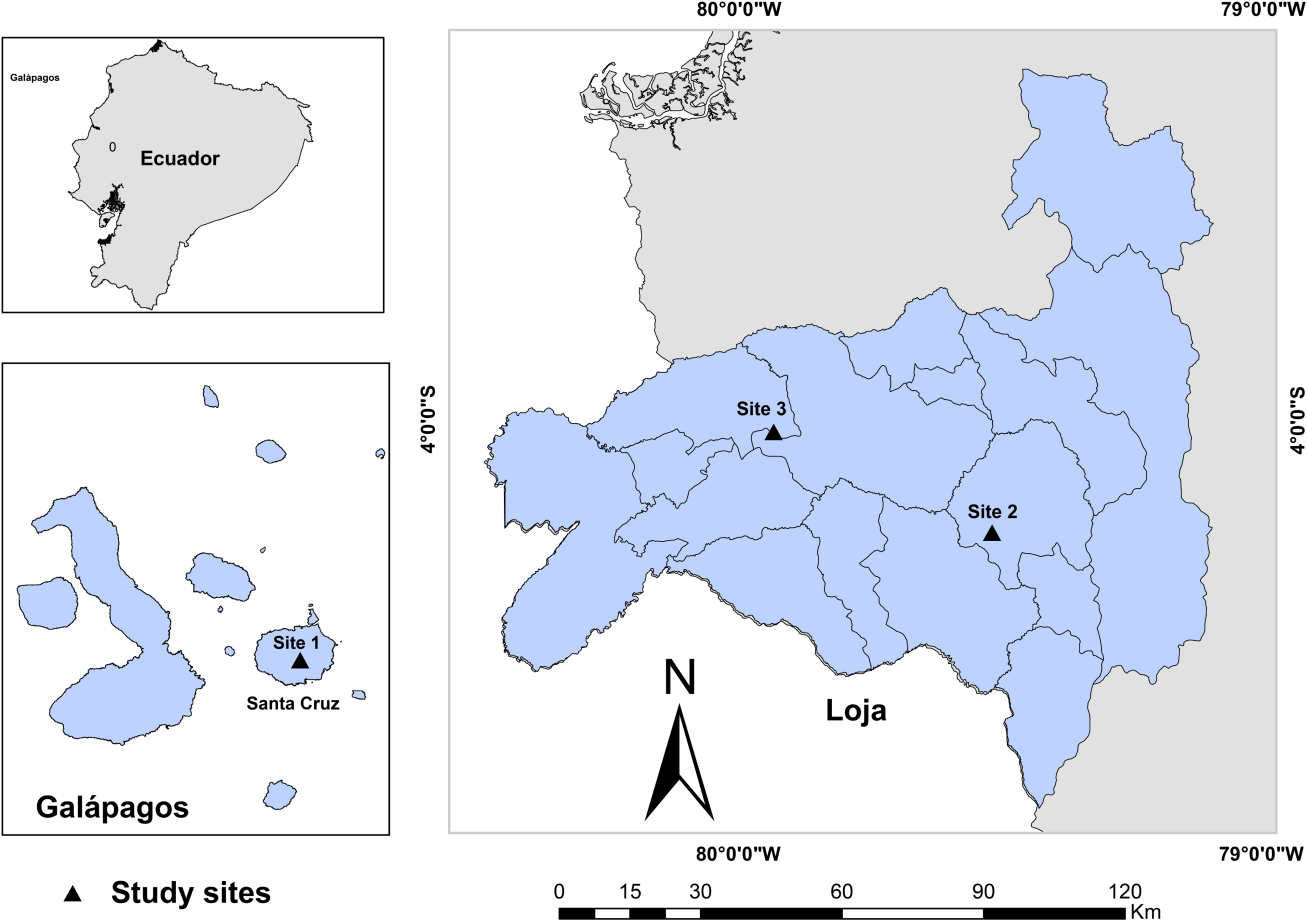
Location of the study sites on Santa Cruz Island (Site 1, Galápagos) and in the Loja province on mainland Ecuador (Site 2 and Site 3).

Site 1 was located in the highlands of Santa Cruz (3°12' S, 85°31' W, at about 610 m a.s.l.), in the Galápagos Islands, Ecuador (Figure 1), the only island invaded by *C. pubescens*. Mean annual precipitation at the study site was about 1700 mm (Hamann 1979), while the annual temperatures varied between 17°C and 33°C (mean minimum and maximum temperature, Jäger, Kowarik, and Tye 2009). The soils were shallow ferruginous andosols, consisting of young pyroclastic deposits with a pH (KCl) of about 4.3–5.2 (Laruelle 1966). This site was originally dominated by a fern‐sedge vegetation (mainly native bracken *Pteridium esculentum* subsp. *gryphus*, other fern species, including the endemic tree fern *Cyathea weatherbyana*, and several herbaceous and gramineous species) and had more recently been invaded by *C. pubescens* and *Rubus niveus* (blackberry) (Jäger, Kowarik, and Tye 2009). *Cinchona pubescens* had become a dominant species here, covering nearly 15% of the area. For further details on the plant species composition, please see Jäger, Kowarik, and Tye (2009).

Sites 2 and 3 were located in the province of Loja, in the South of Ecuador (Figure 1), the only area with an adequate number of remaining *C. pubescens* trees for sampling. Site 2 was a patch of natural forest (4°13' S, 79°31' W, at 1968 m a.s.l.), located in the district of Gonzanamá. Site 3 was a secondary forest, located in the district of Puyango (4°01′25″ S, 79°56′02″ W, at 1920 m a.s.l.) (Figure 1). Both sites were classified as montane cloud forests (Sierra 1999; Valencia et al. 1999). These sites were forest remnants, found on dissected mountains with steep slopes and streams (Gobierno Autónomo Descentralizado Municipal de Puyango [GAD Puyango] 2014), surrounded by grassland, at about 1500 to 2900 m a.s.l. (MAE and FAO 2015; Valencia et al. 1999). Precipitation varied between 700 and 1500 mm per year, with the months of February, March, and April having the highest rainfall (GAD Puyango 2014). The soils of these sites were classified as inceptisol type (Mejía, Maldonado, and Miranda 1986), with the presence of organic matter, and slightly acidic to slightly alkaline pH (GAD Puyango 2014). The canopy of the forest reached 20 m, the understory was dense, and the herbaceous vegetation mainly dominated by ferns, shrubs, and juvenile trees, with many species of bryophytes and epiphytes (orchids, bromeliads, and ferns) (Sierra 1999). Dominant tree species of this ecosystem were *Alnus acuminata* (Betulaceae), *Cecropia maxima* (Cecropiaceae); *Cyathea caracasana* (Cyatheaceae); *Miconia corazonica* (Melastomataceae), *Siparuna guajalitensis* (Siparunaceae), *Chusquea* spp. (Poaceae), *Elaegia utilis*, *Palicourea* spp. (Rubiaceae), and *Nectandra* spp. (Lauraceae) (Sierra 1999). *Cinchona pubescens* trees were scarce and scattered, with about 20 adult individuals per site.

### 2.2 Sampling

Sampling was conducted between 2011 and 2013. Root samples were collected from randomly selected *C. pubescens* trees at 20 different localities in the highlands of Santa Cruz (covering an area of about 400 ha and an altitudinal range from 590 to 620 m a.s.l.) at Site 1. At Sites 2 and 3, samples were collected from 11 trees at each site that were randomly selected at different localities (covering an area of about 1 ha per site). Trees at all three sites were at least 10 m apart from each other, and species identification was based on morphological characters. For sampling, the soil was excavated approximately 40 cm deep in two different areas around the stem base, and clusters of fine roots were sampled by following the stilt roots. Fine roots were preserved in 50% ethanol and stored at 4°C. Additionally, leaves and inflorescences for each sampled individual were collected to confirm the species identification as *C. pubescens*.

### 2.3 Molecular Identification of Fungi

Fungal colonization of root samples was verified according to Beck et al. (2007). Fragments of fine roots (diameter < 2 mm and up to 5 cm in length) were bisected longitudinally and half the sample cleared in 10% KOH for 1 day at 65°C in a water bath. Root of this sub‐sample were then washed twice in tap water, acidified by 10% HCl for 2 min and finally stained with 0.05% methyl blue in 90% lactic acid for 2 h at 65°C. Stained root halves were subsequently analyzed under the microscope (Leitz WETZLAR SM‐Lux), identifying structures as hyphae, vesicles, and arbuscules of AMF. The other half of unstained root halves were used for DNA isolation.

Five to ten colonized root halves per sample were combined and used for DNA isolation (50–100 mg). Total DNA was isolated using the DNeasyPlant Mini Kit (Qiagen, Hilden, Germany), according to the manufacturers' instructions, including a previous step of grinding the root tissue by using an extraction mill (Retsch MM301). A part of the 18 S nrDNA was amplified through a PCR with the Glomeromycotina specific primer pair AML1 and AML2 (Lee, Lee, and Young 2008). The Phusion High‐Fidelity DNA Polymerase 2× Mastermix (Finnzymes, Espoo, Finland) was used for the amplification in a final volume of 20 μL per reaction. PCR conditions were as follows: initial denaturation at 98°C for 15 min; 30 cycles, each cycle consisting of one step of denaturation at 98°C for 10 s; annealing at 60°C for 40 s and extension at 72°C for 1 min and a final extension at 72°C for 10 min was performed to finish the PCR. A control including PCR mix without DNA template was included in every PCR. The success of the PCR amplification was tested in 0.7% agarose stained with Gel Red Nucleic Stain (Biotium, Hayward, CA, USA).

Furthermore, we performed a nested PCR for amplifying samples that could not be amplified in the previous PCR. For that, we used the universal eukaryotic primers NS1 and NS4 (White et al. 1990) in the first PCR and AML1/AML2 in the second one. PCR conditions for the PCR with primers NS1/NS4 were as follows: initial denaturation at 94°C for 3 min; 30 cycles, each cycle consisting of one step of denaturation at 94°C for 30 s; annealing at 40°C for 1 min, extension at 72°C for 1 min and a final extension at 72°C for 10 min.

Cloning of PCR products was performed according to Kottke et al. (2010), using the Zero Blunt TOPO PCR Cloning Kit (Invitrogen, Carlsbad, USA). Per each tree individual, eight clones were selected at random to be sequenced (Macrogen, Seoul, Korea), using the primers M13F and M13R.

### 2.4 Sequence Editing, OTU Delimitation, and Phylogenetic Analyses

Sequences were edited, and consensuses were generated using CodonCode Aligner (CodonCode Corporation, Dedham, MA, USA). Sequences were aligned using the G‐INS‐I option in MAFFT Ver. 6.602b (Katoh et al. 2005). Alignments were checked to eliminate chimeric sequences by manually cutting and comparing unusual part of the sequences on BLAST (< 200 bp by part).

Operational Taxonomic Units (OTUs) were defined based on sequence similarity of the 18S nrDNA region. A table of p‐distance was calculated in PAUP 4.0b10 (Swofford 2002), and grouping of sequences was performed with OPTSIL (Göker et al. 2009) at 99% threshold of sequence similarity and 0,5 of linkage fraction (Haug, Setaro, and Suárez 2013). A representative sequence of each OTU was submitted to GenBank (accession numbers OR707862–OR707897).

A BLAST search for each OTU was performed against the NCBI nucleotide database (GenBank; http://www.ncbi.nlm.nih.gov/), using the option MegaBlast, and also against the MaarjAM database (https://maarjam.botany.ut.ee). Closer sequences from these databases, sequences from Ecuador that showed a similarity of ≥ 99%, and identified Glomeromycota species were added to the analyses and aligned with our sequences, using the G‐INS‐I option in MAFFT v6.602b (Katoh et al. 2005). Neighbor‐joining analyses, using the BioNJ modification with Kimura 2‐distances, were carried out and combined with bootstrap analyses (Felsenstein 1985). Additionally, the BLAST search against the MaarjAM database was used to check the distribution area of the OTUs.

### 2.5 Richness and Similarity of AMF‐OTUs Across Sites

The potential richness and inventory completeness of mycorrhizal OTUs were calculated according to Herrera et al. (2018). Individual‐based species (OTUs) accumulation curves were calculated for each site. These curves and those describing their 95% confidence intervals were fitted to a Clench curve (Soberón and Llorente 1993) following Jiménez and Hortal (2003). The asymptote was also calculated for each curve as a/b, where a and b are parameters of the Clench curve. The proportion of obtained OTUs was calculated according to Jiménez and Hortal (2003).

The similarity in the composition of AMF OTUs between sites was evaluated by similarity indices of Chao‐Jaccard and Chao‐Sørensen based on abundances (Chao et al. 2005), using the program EstimateS Ver. 9.1.0. These indices account for the shared but unobserved OTUs between samples.

Nonmetric multidimensional scaling (NMDS) was used to visualize the differences in AMF OTU community structure. Additionally, analyses of similarity (ANOSIM) was used to test for differences in the composition of OTUs among sites, where the 7*R* statistic varies between −1 and +1, with 0 indicating no separation and +1 complete separation (for interpretation of negative values see Chapman and Underwood 1999). We performed the NMDS and ANOSIM analyses using Bray–Curtis distance and 999 permutations. All analyses were performed using the R package vegan (Oksanen et al. 2017). Additionally, pairwise comparisons of AMF OTU communities between the three sites were performed on PAST Ver. 3.23 (Hammer, Harper, and Ryan 2001).

## 3 Results

### 3.1 Root Colonization by AMF and AMF Sequences

Roots of *C. pubescens* were well colonized by fungal hyphae and vesicles in general, but not so much by arbuscules. We obtained amplicons for 19 samples from Site 1, nine from Site 2, and ten from Site 3. In total, 332 sequences were obtained from all of these amplicons and 211 were discarded due to repeated clones, the presence of chimeras (32 sequences), or high similarity with other organisms distinct from Glomeromycota. Thus, we obtained a total of 121 sequences of the nrDNA 18S belonging to Glomeromycota.

### 3.2 Richness and Similarity of AMF‐OTUs Across Sites

From the three sites, 36 AMF‐OTUs associated with *C. pubescens* were delimited using OPTSIL (a total of 13 additional singletons OTUs were discarded; Table S1). Most of the OTUs belonged to the genus *Glomus* (63.9%), followed by *Acaulospora* (13.9%), *Rhizophagus* (11.1%), *Claroideoglomus* (5.6%), *Archaeospora* (2.8%), and *Gigaspora* (2.8%) (Figure S1).

**FIGURE 2:**
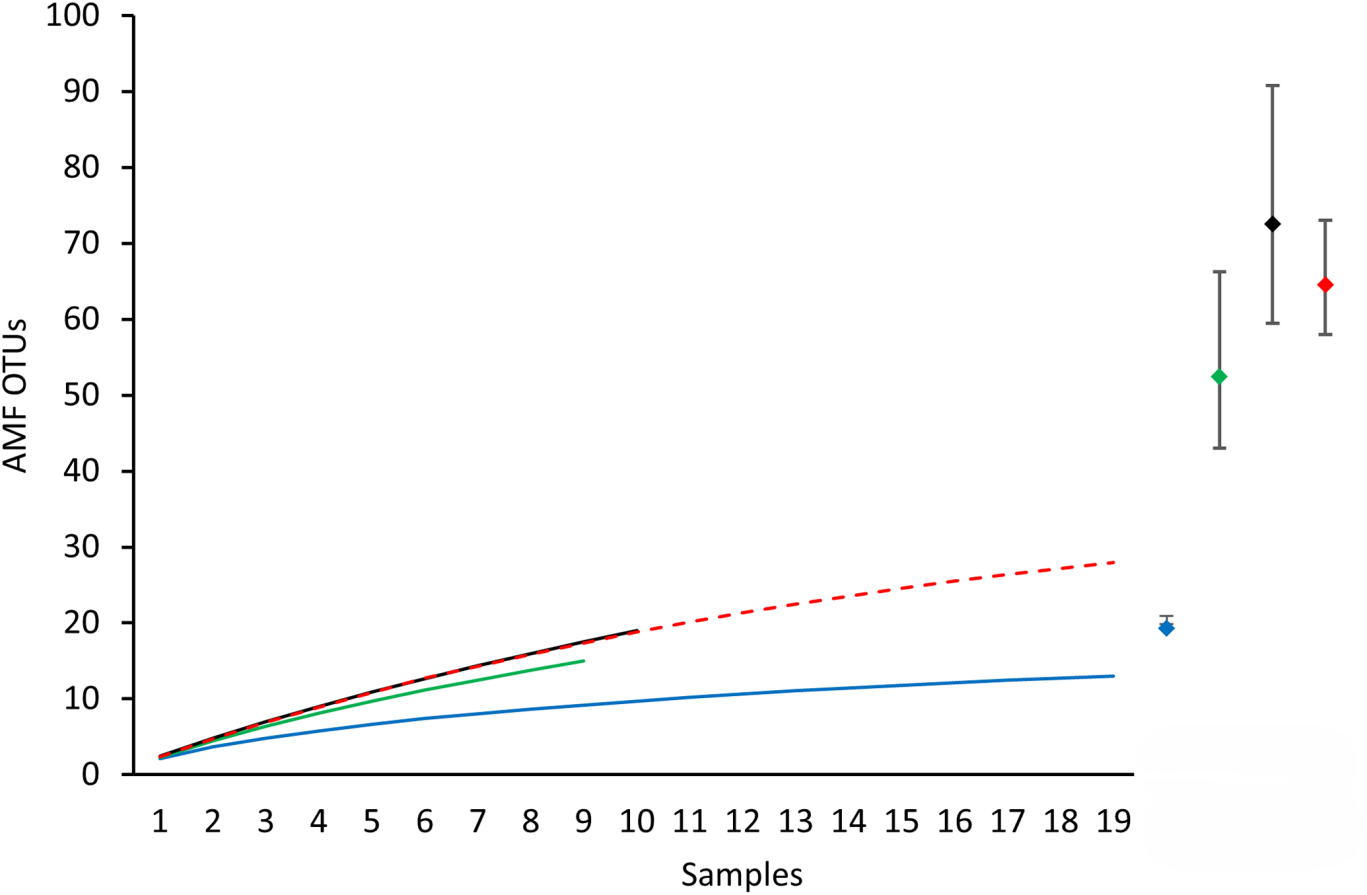
Individual‐based accumulation curves of AMF OTUs associated with *Cinchona pubescens* collected at three sites in Ecuador (Site 1, Santa Cruz: Continuous blue line; Site 2, Loja, natural forest: Green line; Site 3, Loja, secondary forest: Black line; Site 2 plus Site 3: Dashed red line). Diamonds show the asymptotic values for the curves (same colors as curves), and vertical bars indicate their 95% confidence intervals.

Results showed that 13 OTUs were associated with *C. pubescens* at the Galápagos Site 1, 16 OTUs at the Loja Site 2, and 19 OTUs at the Loja Site 3 (Figure 2). Combined samples from the Loja sites showed a significantly higher richness of AMF OTUs associated with *C. pubescens* compared to samples from the Santa Cruz site (Figure 2). There were no significant differences in the OTU richness between samples from the Loja sites (Figure 2). Richness of AMF OTUs was considered significantly different across sites when the 95% confidence intervals of the corresponding accumulation curves did not overlap (Ellison and Gotelli 2004). However, the accumulation curves were asymptotic and the analyses showed an inventory completeness of 67.5% for Site 1, 28.6% for Site 2, and 26% for Site 3 (Figure 2), which indicates that there is a need for further sampling effort to reach the asymptote, and consequently, there may be a greater richness of OTUs associated with *C. pubescens* than detected by our study.

**FIGURE 3:**
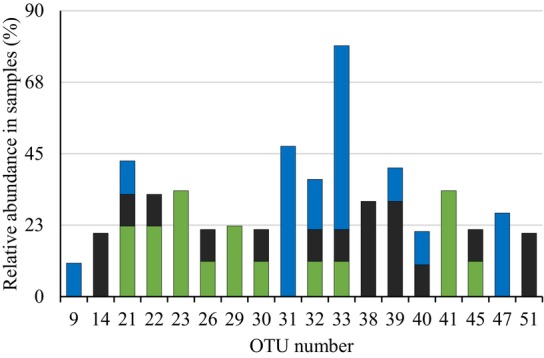
Relative abundance of AMF OTUs found more than once in samples from three sites in Ecuador (Site 1, Santa Cruz: Blue bars; Site 2, Loja natural forest: Green bars; Site 3, Loja, secondary forest; black bars).

Chao‐Jaccard and Chao‐Sørensen indices showed high similarities for the composition of AMF OTUs for *C. pubescens* between Site 2 and Site 3, and a lower similarity between Site 1 and Site 2, and Site 1 and Site 3 (Table 1). Three OTUs associated with *C. pubescens* were shared between all sites, but only two OTUs were exclusively shared between Site 1 and Site 3, and four OTUs between Site 2 and Site 3 (Figure 3). According to BLAST and MaarjAM databases, 12 of the 13 AMF OTUs that were found for *C. pubescens* on Santa Cruz also occurred on mainland Ecuador but associated with other plant species, as shown in Table S2.

**TABLE 1:** Chao‐Jaccard and Chao‐Sørensen similarity indices between the AMF communities of *Cinchona pubescens* at three sites in Ecuador (Site 1: Santa Cruz, Site 2: Loja, natural forest, and Site 3: Loja, secondary forest).

Sites	Shared OTUs observed	Chao‐Jaccard‐Est abundance‐based	Chao‐Sorensen‐Est abundance‐based
Site 1 vs. Site 2	3	0.14	0.24
Site 1 vs. Site 3	5	0.28	0.44
Site 2 vs. Site 3	7	0.97	0.98

At Site 1, OTU 33 was very frequently associated with *C. pubescens*, 58% of the 20 trees had this OTU (Figure 3; Table S2). OTU 33 also occurred at Site 2 and Site 3 (Figure S1), but only in low frequencies. OTU 31 was found to be associated with nine trees (47%) at Site 1 (Figure 3; Table S2; Figure S1) but was neither detected at Site 2 nor at Site 3. However, OTU 31 had previously been found twice to be associated with other plant species in the tropical mountain rainforest in the South of mainland Ecuador, at the Reserva Biológica San Francisco area (Haug, Setaro, and Suárez 2019, 2021) (Table S2). No OTU was dominant at Sites 2 and 3. At Site 2, the highest frequencies were 33% for OTU 41 and OTU 23, and at Site 3, 30% for OTU 38 and OTU 39 (Figure 3; Table S2).

For 22 of the 36 OTUs, we obtained a sequence similarity greater than 99% on the MaarjAM database, confirming the wide distribution of these in other areas of Ecuador and at a global level (Table S2).

**FIGURE 4:**
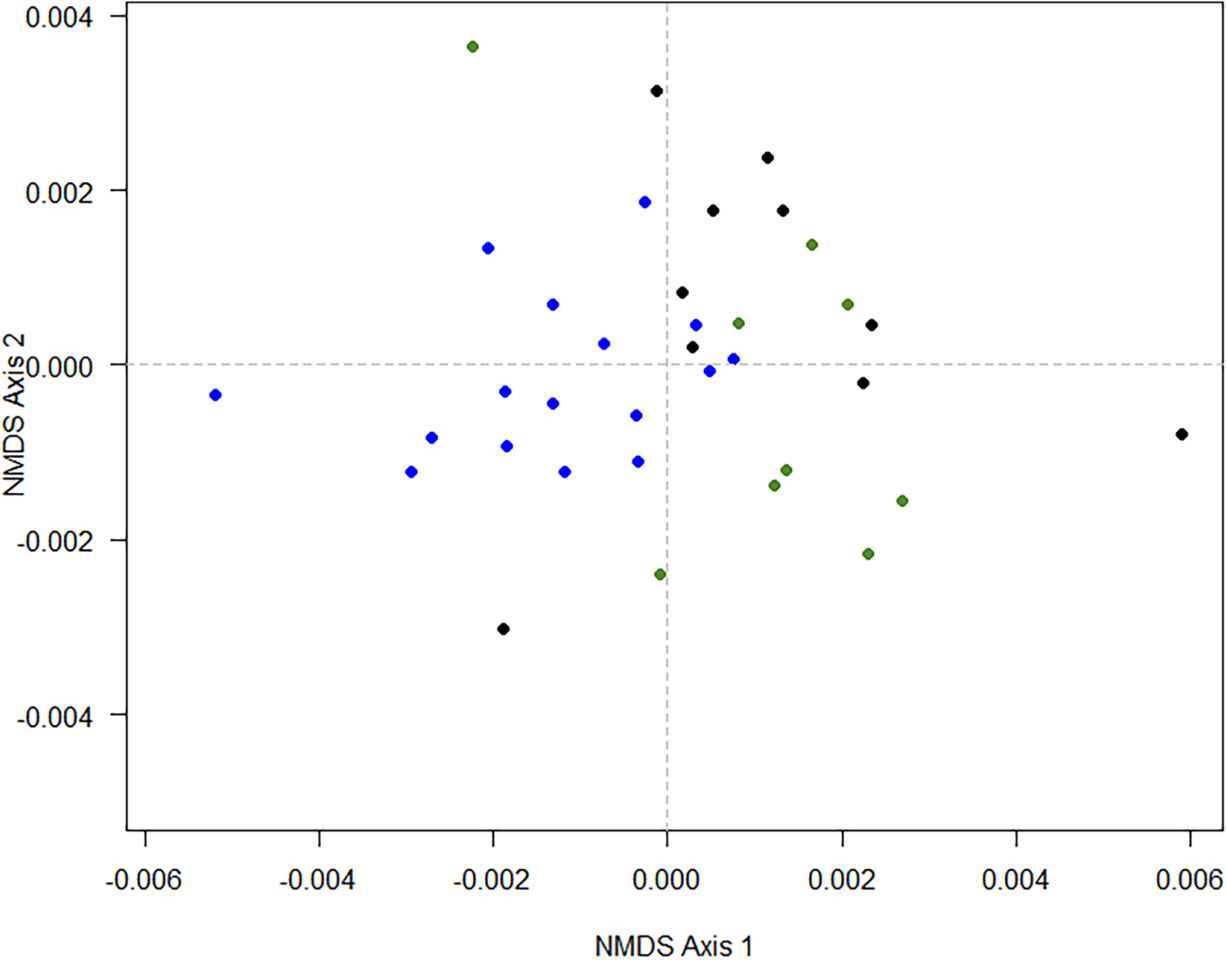
Nonmetric multidimensional scaling plot for AMF OTUs associated with *Cinchona pubescens* based on Euclidean distance across samples from three sites in Ecuador. Blue circle = Site 1, Santa Cruz; green circle = Site 2, Loja, natural forest; and black circle = Site 3, Loja, secondary forest.

The nonmetric NMDS clustering of mycorrhizal OTU data revealed a different pattern between the samples in relation to the site (stress = 0.16, Figure 4). ANOSIM showed a significant effect of the site for the composition of AMF OTU communities (*R* = 0.34, *p* = 0.001). The pairwise test showed that the community at Site 1 was significantly different from Site 2 (*R* = 0.39, *p* = 0.0001) and Site 3 (*R* = 0.32, *p* = 0.0007), but no differences were encountered between Site 2 and Site 3 (*R* = 0.07, *p* = 0.0595).

## 4 Discussion

The vast documentation of AMF diversity associated with different plant species reflects its ecological importance for plant communities, diversity, and productivity in natural ecosystems worldwide (Sanders and Rodriguez 2016; van der Heijden et al. 1998). In the same way, AMF communities play an important role in the successful invasion of new habitats by some plant species due to their ability to promote growth and competition (Chen et al. 2021; Zhang et al. 2018). The underlying mechanisms for this are not fully understood yet, but there is some evidence that AMF enhances the photosynthetic performance of the invasive plant species, as well as its nutrient absorption, especially that of phosphorus (Majewska, Rola, and Zubek 2017; Menzel et al. 2017). Plant species often perform better in their introduced compared to their native range (Parker et al. 2013) due to various factors like soil conditions, climate and environment, growth rates and propagation, among others (Florianová et al. 2023; Maron et al. 2014).

Our results showed that *C. pubescens* was colonized by a total of 36 AMF‐OTUs in its introduced range (Santa Cruz Island, Galápagos) and in its native range (Loja, mainland Ecuador). Most of these OTUs belonged to the genus *Glomus* (63.9%), which is one of the dominant genera of AMF found in various plant species in a wide range of environments, like tropical forests (Kottke et al. 2013), crops (Daniell et al. 2001), grasslands (Vandenkoornhuyse et al. 2002), and on lake islands (Mangan et al. 2004). Apart from *Glomus*, *C. pubescens* was also associated with *Acaulospora*, *Rhizophagus*, *Claroideoglomus*, *Archaeospora*, and *Gigaspora*.

Richness of AMF‐OTUs was higher in *C. pubescens* from the Loja sites compared to the Santa Cruz site, which is in line with the observation that tropical ecosystems usually harbor a high richness of AMF taxa (Kottke et al. 2004; Stürmer and Kemmelmeier 2021). The lower OTUs richness observed on Santa Cruz was similar to a previous study that showed a low AM fungal richness associated with various plant species in Galápagos (Duchicela, Bever, and Schultz 2020).

AMF communities associated with *C. pubescens* from Santa Cruz were significantly different from those associated with *C. pubescens* from Loja, and they only shared five OTUs. Similar differences in AMF communities from island and mainland sites were encountered in Panama (Mangan et al. 2004) but not in a comparison of 13 islands with their respective mainland areas (Davison et al. 2018). The latter finding could be an indication that AMF spores are being transported over longer distances (Bueno and Moora 2019).

Twenty‐two of 36 Glomeromycota OTUs in samples from Santa Cruz and Loja had a wide global distribution (Table S2), which is the case for many taxa of AMF (Davison et al. 2015; Öpik et al. 2013). Since there is anecdotal information that *C. pubescens* was brought to Galápagos from the Loja area of mainland Ecuador (personal communication H. Jäger), it is likely that associated AMF were also introduced. Previous studies have shown that the anthropogenic introduction of microorganisms in soils and plants can result in the dispersal of AMF at large scales (Kivlin, Hawkes, and Treseder 2011; Schwartz et al. 2006). The dispersal of AMF spores to distant oceanic islands is also possible, either by seawater for stress tolerant fungi (Davison et al. 2018) or by transportation through migrating birds (Bueno and Moora 2019). In summary, multiple factors may modulate the AMF diversity and the structure of the AMF community assembly in soil and plants, like the previously mentioned geographic distance, climatic conditions, and plant community type (Kivlin, Hawkes, and Treseder 2011; Lekberg et al. 2007; Rillig et al. 2002). This was also the case for our study sites, with the Santa Cruz site being located more than 1200 km from the Loja sites. Therefore, it is difficult to disentangle the drivers of the differences encountered in the AMF communities between Santa Cruz and mainland Ecuador and further experimental studies should be carried out.

The fact that *C. pubescens* from all three sites were associated with 36 AMF OTUs indicates that there was no specificity in the interaction of *C*. *pubescens* with AMF OTUs. In fact, most of the *C. pubescens* AMF‐OTUs detected were also associated with different plant species in many other areas worldwide (Table S2). AMF are known to being generally low‐specificity mutualists that associate with a wide range of plant species (Smith and Read 2008), and generalist plants tend to associate with generalist AMF (Davison et al. 2011). This was also the case for *C. pubescens* and the local AMF community did not seem to have limited the growth and spread and consequently, the invasion of *C. pubescens* on Santa Cruz Island. Generalists, like *C. pubescens*, usually find their AMF partners among the widely distributed generalist fungal taxa available in the soil in a novel habitat (Moora et al. 2011).

The fact that *C. pubescens* at the two Loja sites had no dominant OTUs, while at Site 1 it was frequently associated with OTU 33 and 31, is striking. It may be that *C. pubescens* has a preference for the OTUs and these then became dominant when the host trees became more abundant as the invasion progressed. However, it is also possible that OTU 33 and 31 were common at this site and therefore preferably associated with *C. pubescens*. AMF studies in Ecuador showed that OTUs can be dominant at certain elevations but not at others in the same area (Haug, Setaro, and Suárez 2019).

The factors that could have facilitated the invasion of *C. pubescens* on Santa Cruz are not well understood and would have to be further examined by experimental approaches. These include niche availability like the fact that the Santa Cruz highlands were formerly treeless and that *C. pubescens* has life history and dispersal traits different from native species (Jäger, Kowarik, and Tye 2009; MacDougall, Gilbert, and Levine 2009; Rundel, Dickie, and Richardson 2014). Some studies showed that AMF have no significant effects on the growth of invasive plant species (Reinhart et al. 2017) and others suggested the opposite (Goodwin 1992; Pringle et al. 2009), and therefore, we cannot rule out that they facilitated the invasion of *C. pubescens* on Santa Cruz Island. Understanding the factors that supported the growth and spread of *C. pubescens* on Santa Cruz Island could help develop conservation strategies for preserving *C. pubescens* on mainland Ecuador, where it is currently rare and endangered due to overexploitation in the past (Günter, Stimm, and Weber 2004).

It is likely that the richness of OTUs associated with *C. pubescens* encountered at both study locations is greater than indicated by our results, since the accumulation curves did not reach the asymptotes. Future studies should increase the sampling effort, especially on mainland Ecuador, where *C. pubescens* trees have become very scarce. Also, for a more accurate characterization of AM fungal communities in *C. pubescens*, the new sequencing techniques (e.g., next‐generation sequencing) (Tedersoo et al. 2010) should be used, including a comparative study of fungal communities in the soils of Galápagos and mainland Ecuador.

In conclusion, our study provides a first comparison of the diversity of AMF associated with *C. pubescens* in its introduced range on Santa Cruz Island, Galápagos, and in its native range in Loja, mainland Ecuador. *Cinchona pubescens* seems to be a generalist that is able to associate with a range of globally distributed AMF. Results showed that *Glomus* was the dominant genus at both locations and that richness of AMF associated with *C. pubescens* from Loja was significantly higher than for *C. pubescens* from Santa Cruz. It seems unlikely that AMF would have acted as a determining factor for the successful invasion of *C. pubescens* on Santa Cruz. However, the presence of dominant AMF OTUs could be an indication that growth and survival of *C. pubescens* were facilitated in its new habitat.

## Author Contributions

**Paulo Herrera:** conceptualization (equal), data curation (equal), formal analysis (equal), writing – original draft (equal), writing – review and editing (equal). **Ingeborg Haug:** conceptualization (equal), data curation (equal), formal analysis (equal), writing – review and editing (equal). **Juan Pablo Suárez:** conceptualization (equal), writing – original draft (equal), writing – review and editing (equal). **Heinke Jäger:** conceptualization (equal), funding acquisition (lead), writing – review and editing (equal).

## Conflicts of Interest

The authors declare no conflicts of interest.

## Supporting information


**Figure S1.** Phylogram inferred from a BioNeighbor‐Joining analysis of partial 18S nrDNA sequences of Glomeromycotina obtained from *C. pubescens* roots with X58724 *Endogone pisiformis* as outgroup. Bootstrap values are given for 1000 replicates, values below 50% are omitted. Sequences were colored according to sampling site as indicated by the key at the top of the tree. The numbers to the right of the tree correspond to the number of OTUs delimited by the 99% threshold.

**Table S1.** Singleton OTUs associated with *Cinchona pubescens* (*C. pub*) at three sites in Ecuador. Numbers in columns D, E, and F indicate the number of observed sequences.

**Table S2.** Occurrence of AMF OTUs associated with *Cinchona pubescens* (*C. pub*) at three sites in Ecuador and closer Virtual Taxa (VT) detected on the MaarjAM database.

## Data Availability

The authors confirm that the data supporting the findings of this study are available within the article and/or its Figure S1 and Tables S1 and S2. Sequences obtained in this study are available in GenBank under accession numbers OR707862–OR707897.
