# Supplementary material for: Globally Distributed Arbuscular Mycorrhizal Fungi Associated With Invasive Cinchona pubescens on Santa Cruz Island, Galápagos
Source: Ecol Evol. 2024 Oct 17;14(10):e70462. doi: 10.1002/ece3.70462 (PMC11483445; doi:10.1002/ece3.70462)
Supplement: Supplementary file 1 — Figure S1. Phylogram inferred from a BioNeighbor‐Joining analysis of partial 18S nrDNA sequences of Glomeromycotina obtained from C. pubescens roots with X58724 Endogone pisiformis as outgroup. Bootstrap values are given for 1000 replicates, values below 50% are omitted. Sequences were colored according to sampling site as indicated by the key at the top of the tree. The numbers to the right of the tree correspond to the number of OTUs delimited by the 99% threshold. [file ECE3-14-e70462-s002.pdf]

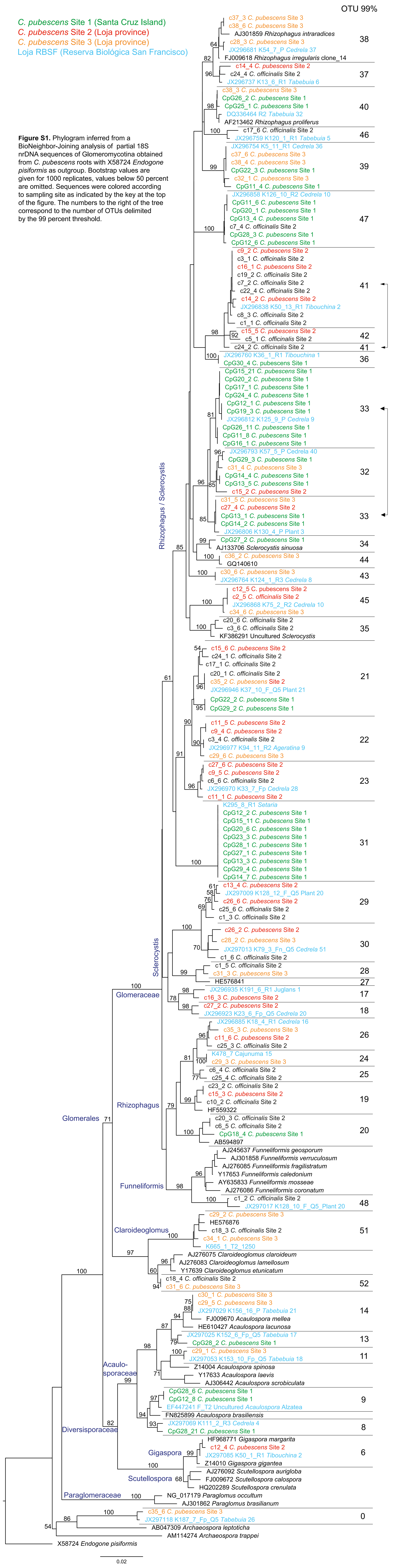

Figure S1. Phylogram inferred from a BioNeighbor-Joining analysis of partial 18S nrDNA sequences of Glomeromycotina obtained from *C. pubescens* roots with X58724 *Endogone pisiformis* as outgroup. Bootstrap values are given for 1000 replicates, values below 50 percent are omitted. Sequences were colored according to sampling site as indicated by the key at the top of the figure. The numbers to the right of the tree correspond to the number of OTUs delimited by the 99 percent threshold.
