# Supplementary material for: Globally Distributed Arbuscular Mycorrhizal Fungi Associated With Invasive Cinchona pubescens on Santa Cruz Island, Galápagos
Source: Ecol Evol. 2024 Oct 17;14(10):e70462. doi: 10.1002/ece3.70462 (PMC11483445; doi:10.1002/ece3.70462)
Supplement: Supplementary file 2 — Table S1. Singleton OTUs associated with Cinchona pubescens (C. pub) at three sites in Ecuador. Numbers in columns D, E, and F indicate the number of observed sequences. [file ECE3-14-e70462-s001.pdf]

Table S1. Singleton OTUs associated with *Cinchona pubescens* (*C. pub*) at three sites in Ecuador. Numbers in columns D, E, and F indicate the number of observed sequences.

OTU id (preliminar analysis)	Cod. Sequence	*C. pub* Loja 1	*C. pub* Loja 2	*C. pub* Galápagos	NCBI			MaarjAM (VT)		
					similarity %	accesion	Organism Filo/ Genus	similarity %	accesion	Organism Family/ Genus/ Species
8	c14_1	1			0.988	MH052365	Uncultured Glomeromycotina	0.971	AJ306437	Gigasporaceae *Scutellospora nodosa* VTX00261
20	c34_1		1		0.999	KF290662	Uncultured Glomeromycota	0.990	EU340324	Claroideoglomeraceae Claroideoglomus NF28 VTX00056
25	c17_5			1	0.965	HF913509	Uncultured Claroideoglomus	0.951	HE615035	Claroideoglomeraceae Claroideoglomus Torrecillas12b Glo G1 VTX00193
30	c28_1		1		0.991	MG829478	Glomeromycotina sp.	0.986	HG004495	Glomeraceae *Glomus* Torrecillas 13 Glo G4 VTX00153
33	c26_4	1			0.989	LS997518	Uncultured *Glomus*	0.986	KC579423	Glomeraceae *Glomus* sp. VTX00304
35	c26_1	1			0.983	FJ831568	Uncultured *Glomus*	0.985	FJ831568	Glomeraceae *Glomus* NF08 VTX00154
47	c12_3	1			0.988	JX296885	Uncultured Glomeromycota	0.983	KJ952224	Glomeraceae *Glomus* sp. VTX00191
53	c11_2	1			0.981	JX296974	Uncultured Glomeromycota	0.978	DQ336505	Glomeraceae *Glomus* Kottke08-13 VTX00219
66	CpG19_1			1	0.998	MG829459	Glomeromycotina sp.	0.991	KJ952238	Glomeraceae *Glomus* sp. VTX00126
74	c28_6		1		0.995	FJ831523	Uncultured *Glomus*	0.995	FJ831527	Glomeraceae *Glomus* NF02 VTX00248
76	CpG23_1			1	0.981	MG829381	Glomeromycotina sp.	0.976	FR821553	Glomeraceae *Glomus* sp. VTX00363
78	c34_5		1		0.971	KX108302	Uncultured Glomeromycotina	0.965	EU417582	Glomeraceae *Glomus* *Afrothismia gesnerioides* symbiont VTX00076
88	CpG18_1			1	0.959	HG380140	Uncultured *Glomus*	0.954	DQ396749	Glomeraceae *Glomus* PF13 VTX00191
