# Supplementary material for: Globally Distributed Arbuscular Mycorrhizal Fungi Associated With Invasive Cinchona pubescens on Santa Cruz Island, Galápagos
Source: Ecol Evol. 2024 Oct 17;14(10):e70462. doi: 10.1002/ece3.70462 (PMC11483445; doi:10.1002/ece3.70462)
Supplement: Supplementary file 3 — Table S2. Occurrence of AMF OTUs associated with Cinchona pubescens (C. pub) at three sites in Ecuador and closer Virtual Taxa (VT) detected on the MaarjAM database. [file ECE3-14-e70462-s003.pdf]

Table S2. Occurrence of AMF OTUs associated with *Cinchona pubescens* (*C. pub*) at three sites in Ecuador and closer Virtual Taxa (VT) detected on the MaarjAM database.

OTU id	GenBank accession	*C. pub* Loja 1	*C. pub* Loja 2	*C. pub* Galápagos	Occurrence in Ecuador *	Worldwide occurrence	species genus	NCBI 99% MaarjAM 99% (VT)	MaarjAM
0	OR707862		1				*Archaeospora*		
6	OR707895	1					*Gigaspora*	**VT39**	175 seq/worldwide/many biomes
8	OR707896			1			*Acaulospora*		
9	OR707897			2			*Acaulospora*	**VT14**	89 seq/several countries/forest, grassland, shrubland
11	OR707863		1				*Acaulospora*		
13	OR707864			1			*Acaulospora*		
14	OR707865		2				*Acaulospora mellea*	**VT24**	124 seq/worldwide/many biomes
17	OR707866	1					*Glomus*	**VT166**	867 seq/worldwide/many biomes
18	OR707867	1					*Glomus*		
19	OR707868	1					*Glomus*		
20	OR707869			1			*Glomus*		
21	OR707870	2	1	2			*Glomus*		
22	OR707871	2	1				*Glomus*	**VT219**	370 seq/worldwide/many biomes
23	OR707872	**3**					*Glomus*		
24	OR707873		1				*Glomus*	**VT191**	535 seq/worldwide/many biomes
26	OR707874	1	1				*Glomus*		
28	OR707875		1				*Glomus*		
29	OR707876	2					*Glomus*		
30	OR707877	1	1				*Glomus*		
31	OR707878			9			*Glomus*		
32	OR707879	1	1	3			*Glomus*	**VT269**	8 seq/mainly tropical/tropical forest, grassland
33	OR707880	1	1	11			*Glomus coremioides*	**VT268**	19 seq/mainly tropical/tropical forest
34	OR707881			1			*Glomus sinuosum/Sclerocystis sinuosa*	**VT69**	218 seq/worldwide/many biomes
36	OR707882			1			*Glomus*		
37	OR707883	1					*Rhizophagus*	**VT112**	212 seq/worldwide/many biomes
38	OR707884		**3**				*Rhizophagus irregularis*	**VT113**	1150 seq/worldwide/many biomes
39	OR707885		**3**	2			*Rhizophagus*	**VT280**	232 seq/worldwide/many biomes
40	OR707886		1	2			*Rhizophagus proliferus*	**VT99**	52 seq/several countries/ forest, grassland
41	OR707887	**3**					*Glomus*		
42	OR707888	1					*Glomus*		
43	OR707889		1				*Glomus*	**VT84**	111 seq/worldwide/many biomes
44	OR707890		1				*Glomus*	**VT309**	41 seq/several countries/ anthropogenic
45	OR707891	1	1				*Glomus*	**VT223**	39 seq/several countries/ many biomes
47	OR707892			5			*Glomus*	**VT68**	5 seq/4 countries/tropical forest
51	OR707893		2				*Claroideoglomus*	**VT56**	443 seq/worldwide/many biomes
52	OR707894		1				*Claroideoglomus lamellosum*	**VT193**	818 seq/worldwide/many biomes
**OTUs total**		**16**	**19**	**13**					
samples		9	10	19					

* data from Haug et al. (2010, 2013, 2019, 2021).
